# Effects of electroacupuncture on the kisspeptin-gonadotropin-releasing hormone (GnRH) /luteinizing hormone (LH) neural circuit abnormalities and androgen receptor expression of kisspeptin/neurokinin B/dynorphin neurons in PCOS rats

**DOI:** 10.1186/s13048-022-01078-x

**Published:** 2023-01-17

**Authors:** Ge Xu, Xuedan Zhao, Zhihao Li, Junwei Hu, Xu Li, Juanjuan Li, Yuelai Chen

**Affiliations:** 1 Longhua Hospital Affiliated to Shanghai University of Traditional Chinese Medicine, Shanghai, 200032 China; 2 Shanghai University of Traditional Chinese Medicine, Shanghai, 201210 China

**Keywords:** Polycystic ovary syndrome, Kisspeptin, Gonadotropin releases hormone, Androgen receptor, Electroacupuncture

## Abstract

**Background:**

Polycystic ovary syndrome (PCOS) is characterized by hyperandrogenism, anovulation, and polycystic ovaries. Electroacupuncture (EA) can effectively improve hyperandrogenism and increase ovulation frequency in patients with PCOS. Pieces of suggest that androgen activity in the brain is associated with impaired steroid negative feedback in such patients. Studies have shown that EA regulated androgen receptor (AR) expression and local factor levels (such as anti-Müllerian hormone and inhibin B) in the ovary of PCOS rats. However, few studies have explored the effect of EA on androgen activity in the brain.

**Objective:**

This study investigated the effect of EA on the kisspeptin-gonadotropin-releasing hormone (GnRH)/luteinizing hormone (LH) neural circuit and sex hormone receptor expression in the hypothalamus of PCOS rats.

**Methods:**

PCOS signs were induced by letrozole administration, and the induced rats were treated with low-frequency EA at Guan Yuan acupoint (CV4). The effect of EA on PCOS-like signs was evaluated by observing changes in the body weight, ovarian quality, ovarian morphology, and serum sex hormone levels in rats. To explore the mechanism of the effect of EA on PCOS-like signs, the neuropeptide content of the kisspeptin-GnRH/LH neural circuit was assessed using enzyme-linked immunosorbent assay(ELISA); AR and estrogen receptor α (ERα) coexpression on kisspeptin/neurokinin B/dynorphin (KNDy) neurons was determined via triple-label immunofluorescence; and protein and mRNA expression of *Kiss1*, *Ar*, *Esr1*, and *kisspeptin receptor* (*Kiss1r*) was evaluated via western blotting and Reverse Transcription-Polymerase Chain Reaction (RT-PCR).

**Results:**

The results revealed that the estrous cycle of rats in the EA treatment group recovered, and their body and ovary weight reduced; ovarian morphology improved; serum testosterone and LH levels significantly decreased; and kisspeptin, GnRH, and dynorphin levels in hypothalamic arcuate nucleus significantly decreased. Compared with controls, the number of AR/Kiss1-positive cells increased, number of ERα/Kiss1-positive cells decreased, and protein and mRNA expression of *Kiss1*, *Ar*, and *Kiss1r* significantly increased in PCOS rats. However, EA treatment reversed these changes and reduced the expression of *Kiss1*, *Ar*, and *Kiss1r* significantly.

**Conclusion:**

Improvement in the reproductive hallmarks of PCOS rats via EA may be achieved by regulating the kisspeptin-GnRH/LH circuit via androgen activity attenuation. Thus, the results provide an experimental basis for acupuncture as an adjuvant medical therapy on PCOS.

## Introduction

Polycystic ovary syndrome (PCOS) is a group of endocrine and metabolic disorders with complex and diverse pathogenic causes and heterogeneous clinical manifestations. It is characterized by infertility, polycystic ovaries, oligo-ovulation/anovulation, hyperandrogenism, and elevated serum luteinizing hormone (LH) levels. Depending on the criteria, the reported PCOS incidence ranges from 6 to 20% in different areas [1–4], making it the most common endocrine disease in women of reproductive age. The pathogenesis of PCOS remains unclear, and current treatments, which rely on the empirical treatment of symptoms, are not ideal, and mechanism-based therapy remains out of reach. Electroacupuncture (EA), derived from traditional Chinese medicine (TCM), can effectively improve hyperandrogenism and insulin resistance [5], increase ovulation frequency, and improve follicular growth in patients with PCOS [6–9]. However, the exact mechanism through which EA works is still unclear.

Increased LH pulse frequencies are commonly reported in women with PCOS [10]. This pattern indicates the increased pulse frequency stimulation by gonadotropin-releasing hormone (GnRH) and increased activity of GnRH neurons upstream in the hypothalamus [11]. Compared with healthy women, patients with PCOS require higher concentrations of exogenous estradiol (E_2_) and progesterone to slow their high-frequency pulsatile LH release [12, 13]. This suggests an impaired ability of steroid hormones to inhibit GnRH/LH pulsatility. Evidence suggests that inappropriately high androgen levels are associated with impaired steroid negative feedback in patients with PCOS. Long-term flutamide treatment can rebuild the ability of E_2_ and progesterone to supperess LH pulse frequency, restore steroid feedback sensitivity, and increase ovulatory cycles [14]. However, the acute administration of testosterone (T) to healthy controls did not increase pulsatile LH levels [15], whereas in patients with PCOS, the administrat-ion of the androgen receptor (AR) antagonist flutamide did not immediately restore normal LH secretion [14]. These findings suggest that androgen excess does not directly drive LH hypersecretion but rather interferes with the ability of E_2_ and progesterone to transmit negative feedback signals to GnRH neurons and inhibit GnRH pulsation, which indirectly leads to the increase of GnRH pulsatility and excessive LH secretion by the pituitary.

The activity of GnRH neurons and pulsating pattern of GnRH secretion are highly dependent on the homeostatic feedback of sex hormone signals in the brain, and the abnormal secretion of GnRH neurons indicates impaired feedback regulation of sex hormones. GnRH neurons only express estrogen receptor β and not the classical estrogen receptor α (ERα), progesterone receptor (PR), or AR [16, 17], and sex hormone-mediated feedback regulation is largely indirect and facilitated by neuronal network located upstream of GnRH neurons.

There is increasing evidence suggesting that kisspeptin/neurokinin B/dynorphin (KNDy) neurons, which are located in the arcuate nucleus (ARC), are associated with hyperactive GnRH/LH pulses in PCOS [18–21]. KNDy neurons secrete kisspeptin that regulates GnRH neurons via the kisspeptin-Kiss1r (Kiss1r) signaling pathway, which in turn regulates the hypothalamic–pituitary–gonadal (HPG) axis function [22, 23]. Most KNDy neurons colocalize ERα, PR, and AR [24, 25], consistent with the view that these neurons are key targets for the actions of sex steroids in the adult brain. KNDy neurons simultaneously secrete neurokinin B (NKB) and dynorphin (Dyn) to regulate the pulsatile release of kisspeptin in an autocrine or paracrine manner [18]. Kisspeptin, encoded by *Kiss1*, is the most potent stimulator of gonadotropin secretion in mammals and is essential for the initiation of puberty, maintenance of normal adult reproductive functions, and coordinating sex steroid feedback in the reproductive axis [11].

Pieces of evidence from both human and animal models indicated abnormal kisspeptin secretion in PCOS. In patients with PCOS, the serum kisspeptin level was increased [26, 27] and positively correlated with the LH [21, 28] and free T levels [29]. In several animal models of PCOS, KNDy expression and circuitry are subtly altered. Studies have found that prenatal dihydrotestosterone exposure in rats leads to elevated LH, which is associated with a minor increase in the number of kisspeptin and NKB-immunoreactive neurons in ARC [30]. In prenatally T-treated ewes, Dyn and NKB immunorea-ctivity in KNDy neurons was reduced, but kisspeptin expression remained unchanged [31]. In a letrozole-induced PCOS model, kisspeptin receptor mRNA increased slightly in the rostral forebrain region containing GnRH neurons, suggesting that GnRH neurons are more sensitive to kisspeptin signaling [32]. The activation of Kiss1 neurons in hypothalamic ARC was promoted in letrozole-treated female mice [33].

In a recent study, control mice with experimental PCOS induced via androgen excess displayed many PCOS traits. However, the superimposition of transgenic AR resistance, in which AR actions were silenced either in the brain or in ovarian granulosa cells, revealed that only female mice with the loss of AR function in the brain were protected from developing the majority of reproductive and metabolic PCOS traits [34, 35]. These findings pinpoint the brain as the potential site at the core of PCOS pathogenesis and strongly support neuroendocrine androgen-driven molecular mechanisms as key mediators in the developmental origins of PCOS traits. The treatment of patients with PCOS with the antiandrogen flutamide has been reported to restore menstrual regularity and ovulation [36]. Moreover, antiandrogen therapy improved reproductive and metabolic phenotypes in letrozole-treated female mice [37]. Therefore, central AR blockade and KNDy regulation may be the upstream targets to improve the activity of GnRH/LH pulse generator. One important question is whether EA affects the regulation of androgens on KNDy neurons.

A previous study revealed that EA can effectively restore the estrous cycle, improve the ovarian polycystic morphology, regulate circulating sex hormone levels, improve insulin resistance, and increase ovarian AR expression in PCOS rats [38]. Although another study showed that EA reduced the expression of kisspeptin in the hypothalamus and ARC of an adolescent rat PCOS model [39], it is unclear whether the effect of EA is related to the effect of androgen inhibition and impairment of PCOS steroid hormone feedback. Thus, the present study aimed to reconfirm the effect of EA on the hypothalamic kisspeptin/GnRH/LH neural circuit of PCOS rats with letrozole models and explore whether this effect is realized by weakening the androgen activity in the brain and restoring the normal steroid hormone feedback mechanism. The results may help clarify the central neuroendocrine pathological mechanism of PCOS, elucidate the experimental basis for the therapeutic effect of EA, and develop new therapeutic methods.

## Materials and methods

### Animals

Sixty 6-week-old female SD rats, with a body weight of 160 ± 20 g were purchased from Beijing Weitong Lihua Laboratory Animal Co., Ltd. (Beijing, China). The rats were housed in the laboratory animal center of Shanghai University of Traditional Chinese Medicine under controlled lighting (12 h light and 12 h dark). All rats were provided with ad libitum access to food and water in a room with a constant temperature (22 °C ± 1 °C) and humidity (65–70%). The animal experiments were approved by the Animal Ethical Committee of Shanghai University of Traditional Chinese Medicine (PZSHUTCM21070200114).

### Study procedure

After 3 days of adaptive feeding, the rats were randomly classified into the following three groups (*n* = 20 per group): control (CON) group, letrozole (LE) group, and EA group. According to the Kafali method [40], PCOS-like traits in rats were induced through daily gavage with 1% sodium carboxymethylcellulose (CMC)-letrozole (H20133109, Zhejiang Hisun Pharmaceutical Co., Ltd., China) suspension (10 mL/kg/d) for 21 consecutive days. CON rats were gavaged with equal volume 1% CMC (20,201,111, Sinopharm Chemical Reagent Co., Ltd., China) solution for 21 days. Starting on the 12th day of letrozole administration, the estrous cycle of rats was observed for 24 consecutive days via vaginal smear to determine whether the modeling was successful and to assess the impact of treatment.

**Fig. 1:**
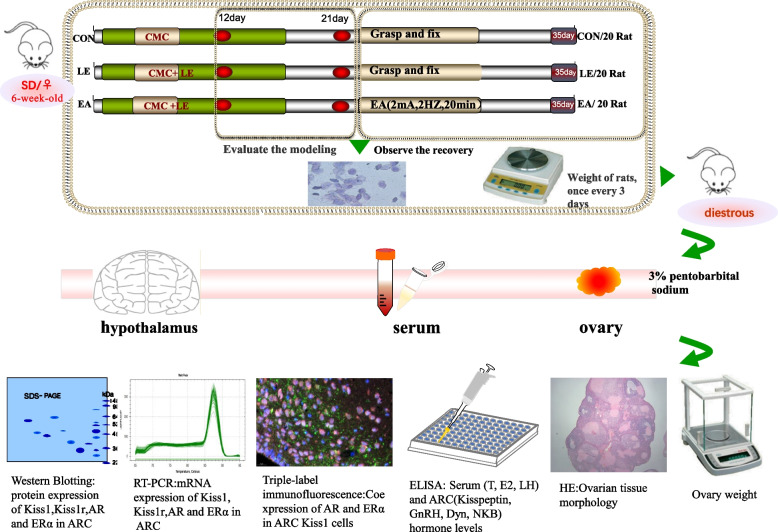
Experimental process

Starting on the 2nd day after modeling, the EA group received EA treatment at Guan Yuan acupoint (CV4) for 14 days. The CON and LE groups were untreated, except for fixation. The body weight of the rats was weighed every 3 days, and their survival status was observed (Fig. 1).

### EA treatment

The rats in the EA group were grabbed and held using a fixator every day, and CV4 (on the ventral midline at approximately the upper 3/5 and lower 2/5 of the line) and the point 5 mm next to CV4 at the same horizontal axis were used. For EA, 1-in. acupuncture needles (Φ0.25 × 25 mm) were inserted to a depth of 3–5 mm and attached bilaterally to Hwato SDZ-II EA therapeutic apparatus (Suzhou medical appliance factory, Suzhou, China). The points were electrically stimulated using a low frequency of 2 Hz and adjusting the intensity to 2 mA; the retention time was 20 min. Rats were treated daily for 14 consecutive days. The complete experimental process is shown in Fig. 1.

### Blood and tissue collection

On the 2nd day after EA treatment, vaginal smears were examined before sampling at 7:00 am. Blood as well as the ovary and hypothalamus tissue samples of rats during diestrus were collected. Rats were first anesthetized with 3% pentobarbital sodium (0.1 mL/100 g of body weight), and blood samples were obtained from the abdominal aorta. The blood samples were centrifuged at 3000×*g* for 15 min at 4 °C, and serum was stored at − 80 °C until hormone analysis. The left ovaries of rats in each group were removed. After the surrounding tissues were removed and weighed, 10 samples of each group were fixed with 4% paraformaldehyde (PFA). In addition, 10 samples of each group were frozen in liquid nitrogen and then transferred to − 80 °C refrigerator for subsequent protein and gene expression detection. Five rats in each group were perfused with normal saline and 4% PFA sequentially. Their brains were then quickly separated after the tissue became white and postfixed in 4% PFA for 24 h at 4 °C. The complete brains of the remaining 15 rats in each group were placed on ice, and their hypothalamic ARC was quickly isolated by referring to the stereotaxic map of the rat brain and stored in a − 80 °C refrigerator. Considering the middle of the tuber cinereum and optic chiasma as the center, prechiasmal border as the anterior edge, and postmammillary border as the posterior edge, the hypothalamus (4 × 4 × 2 mm) was carefully removed. ARC (located 2.0–4.5 mm posterior to the bregma and 0.5 mm beside the mid raphe near the median eminence) was carefully excised and collected.

### Vaginal smear and estrous cycles

Vaginal smears were prepared daily at 08:00 am from the 12th day to the end of the study. Vaginal smear microscopy (BX41, Olympus, Japan) was performed to determine the stage of the estrous cycle via the predominant cell types following Ray–Giemsa staining (Nanjing Jiancheng Biological Engineering Institute, Nanjing).

### Morphological observation

Fresh ovarian tissues were fixed in 4% PFA for 24 h, routinely dehydrated, embedded in paraffin, and sliced at 150–200-μm intervals with a slice thickness of 4-μm. Five slices from the same ovary were mounted on a glass slide and baked in a 60 °C oven. After dewaxing and hydration, the sections were stained with hematoxylin for 15 min, differentiated with 1% hydrochloric acid and ethanol, stained with 1% eosin solution, dehydrated to transparency, and sealed with neutral gum. The morphology of the ovarian tissue sections was examined using Leica DM 2000 microscope (Leica, Wetzlar, Germany) at 40×, 100×, and 400× to analyze the characteristics of follicle granules and membrane cell layer of ovarian follicles at different stages. The number of cystic follicles and corpora lutea in the hematoxylin and eosin-stained sections of rat ovaries was counted in the low-power field (10×) by two researchers to avoid duplicate counting.

### Enzyme-linked immunosorbent assay (ELISA)

The serum concentrations of T, E_2_, and LH and hypothalamic ARC levels of kisspeptin, GnRH, Dyn, and NKB were quantified using rat ELISA kits (Shanghai Hengyuan Biological Co., Ltd., Shanghai, China). All experiments were performed according to the manufacturer’s instructions. The kit detection range was 5–160 nmol/L for T, 3–80 ng/L for E_2_, 1.5–40 ng/L for LH, 10–350 pg/mL for kisspeptin, 1–48 ng/L for GnRH, 0.2–9 μg/L for NKB, and 12–500 pg/mL for Dyn.

### Triple-label immunofluorescence of kisspeptin, AR, and ERα

After being fixed in 4% PFA for 24 hours, the brain tissues were then incubated in 30% sucrose prepared in Tris buffer saline solution for 48 h at 4 °C. Serial coronal sections (20 μm) were prepared from the bregma anterior–posterior from − 2.0 to − 3.30 mm. Five tissue sections from each group of rats were selected for Kiss1, ERα, and AR immunofluorescence staining. The sections (4 μm/slide) were deparaffinized, hydrated, and pretreated in a microwave (antigen retrieval). After antigen retrieval, the sections were sealed and incubated with serum. Primary antibodies against ERα (1:50, Novus, USA), AR (1:50, Novus, USA), and Kiss1 (1:100, Thermo Fisher, USA) were added and incubated at 4 °C overnight. The samples were washed with phosphate-buffered saline(PBS) and incubated for 1 h at 4 °C with the following fluorescent secondary antibodies: CY3-donkey anti-mouse IgG H&L (Alexa Fluor® 405) (1:300, Abcam, UK) + donkey anti-goat IgG H&L (Alexa Fluor® 647) (1:500, Abcam, UK) + donkey anti-rabbit IgG H&L (FITC) (1:100, Abcam, UK). After removing the bound primary and secondary antibodies, the nuclei were counterstained with DAPI staining solution and mounted after incubation. The sections were observed under a fluorescence microscope (Nikon ECLIPSE C1, Japan), and images were captured. The Halo v3.0.311.314 image quantitative analysis system (Indica labs, USA) was used to quantify the expression of AR, ERα, and Kiss1 in the target area of each section.

### Western blotting

The hypothalamic ARC tissues of rats in each group were homogenized using frozen RIPA lysis buffer and protease inhibitors. After ensuring complete tissue lysis, the solution containing total proteins was obtained by centrifugation, and the protein concentration was determined using the BCA protein detection kit (Thermo Fisher, USA). Electrophoresis was performed, and the separated samples were transferred via a membrane. To this membrane, the primary antibody solutions of Kiss1 (1:1000, Thermo Fisher, USA), Kiss1r (1:1000, Thermo Fisher, USA), AR (1:1000, Novus, USA), and ERα (1:1000, Novus, USA) were added and incubated overnight at 4 °C. Then, the secondary antibody donkey anti-goat IgG-H&L (1:3000, Abcam, UK) was added, and the protein antibody complexes were visualized using the ECL Chemiluminescence Visualization Kit (Servicebio, Wuhan, China). After the exposed film was developed and fixed, it was arranged using PhotoShop. The Alpha software (Innotech alphaEaseFC, USA) processing system was used to analyze the gray value of the target protein. The relative expression of Kiss1, Kiss1r, AR, and ERα was calculated by comparing the gray value ratio to the internal reference glyceraldehyde-3-phosphate dehydrogenase (GAPDH).

### mRNA isolation and real-time reverse transcription-polymerase chain reaction (RT-PCR)

Total RNA was isolated using the TRIzol reagent (Invitrogen, Carlsbad, CA, USA). Nanodrop 2000 was used to detect the concentration and purity of RNA, which was then diluted in an appropriate proportion to make the final concentration of 100–500 ng/μL. Total RNA (20 μL) was reverse transcribed to cDNA using the Superscript Reverse Transcriptase kit (Takara, Japan). Finally, real-time PCR was performed with TB Green™ Premix Ex Taq™ (Takara, Japan) using a fluorescence quantitative PCR instrument (Bio-Rad-CFX, US) and SDS software (Applied Biosystems, Foster City, CA, USA). Primers were procured from Servicebio (Servicebio, Wuhan, China), and the sequences are shown in Table 1. *Gapdh* was considered as the housekeeping gene to normalize the expression levels of *Kiss1*, *Kiss1r, Ar, Esr1*. The PCR amplification conditions were as follows: initial denaturation at 95 °C for 30 s and amplification for 40 cycles with denaturation at 95 °C for 15 s, annealing at 60 °C for 30 s, and extension at 60 °C for 30 s. The reaction was followed by melt curve analysis to determine homogeneity of the amplicons. The fluorescence signal was collected every 0.5 °C during the reaction process from 65 °C to 95 °C. All experiments were performed in triplicate, and the relative expression of genes was analyzed according to the 2 ^−∆∆Ct^ method.

**Table 1:** Primer sequences of the targeted genes in rats

Targeted genes	Forward and reverse primers (5′ → 3′)	Amplicon size (bp)	Accession number
*Kiss1*	TGTCAGCCTACAACTGGAACTCC	147	NM_181692.1
	TTGCACAAGTCTAGAAGCTCCCT		
*Kiss1r*	TAGTCGGGAACTCACTGGTCATC	241	NM_001301151.1
	CTCATGGCTGTCAAAGTGGCA		
*Ar*	CTGATTCCTTTGCTGCCTTGT	188	NM_012502.2
	ATTAGTGAAGGACCGCCAACC		
*Esr1*	TAGTCGGGAACTCACTGGTCATC	178	NM_012754.3
	CTCATGGCTGTCAAAGTGGCA		
*Gapdh*	CTGGAGAAACCTGCCAAGTATG	138	NM_017008.4
	GGTGGAAGAATGGGAGTTGCT		

### Statistical analysis

Data were analyzed using the SPSS 25.0 software. Experimental data are expressed as mean ± standard deviation. All data were normally distributed, and one-way analysis of variance was used for comparison between groups. The least significant difference test was used for the pairwise comparison of homogeneity of variance, and Dunnet’s test was used for evaluating the heterogeneity of variance. *P* < 0.05 was considered significant.

## Results

### Improvement in estrous cyclicity

**Fig. 2:**
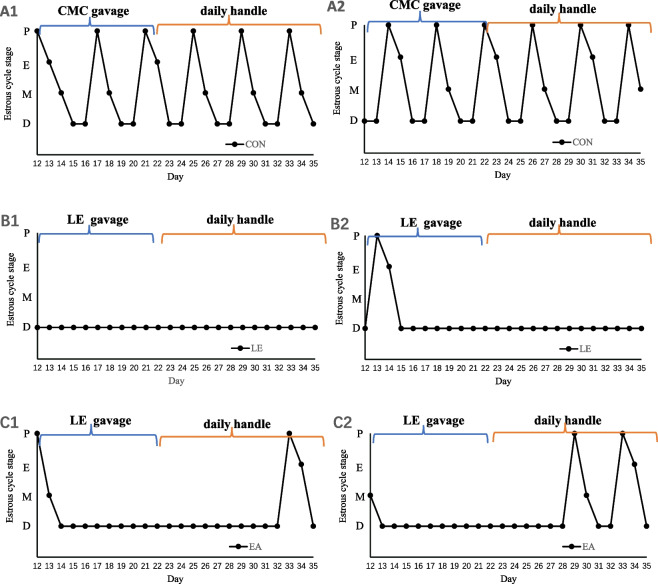
Representative estrus cycles at 12-35d of rat from the CON, LE, and EA groups. (*n* = 20 rats/group). From the 12th day of LE administration to the end of the experiment, the rats in PCOS group showed persistent diestrus, whereas rats in EA group gradually showed estrous cyclesduring during the treatment period. P, proestrous. E, estrous. M, metestrous. D, diestrous

To determine the success of modeling, the estrous cycle was assessed via the microscopic analysis of major cell types in the vaginal smears obtained daily for 12 days after letrozole gavage. All rats in the CON group showed normal estrous cyclicity. A total of 20% (8 of 40) rats in the LE group began losing the normal estrous cycle on the 12th day after letrozole administration, and by the 15th day, all rats showed persistent diestrus. The estrus cycle of 25% (5 of 20) rats in the EA group recovered on the 8th day of treatment, and by the 12th day, the estrous cycles of all rats had recovered. The rats in the LE group were still in the diestrus stage at the time of euthanasia. The representative cycles of each group are shown in Fig. 2.

### Rat body weight

**Fig. 3:**
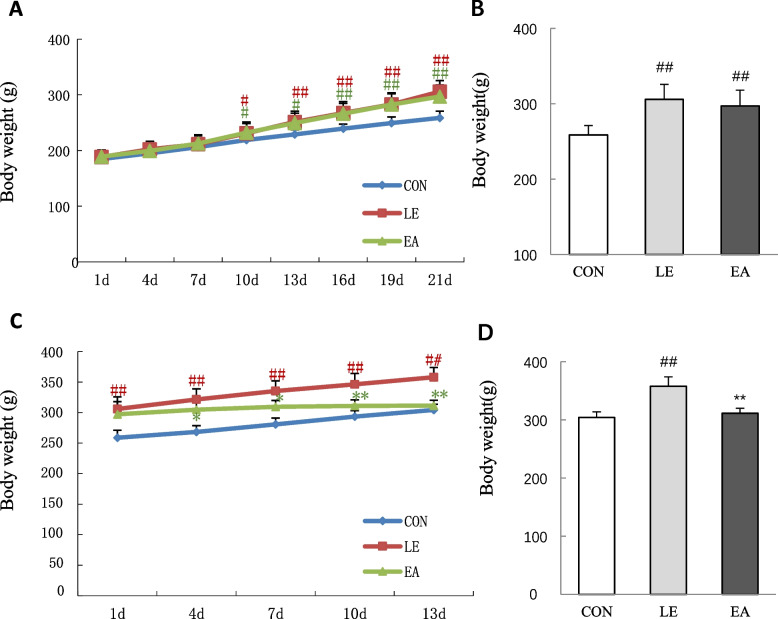
Body weight of rats during the experimental period (*n* = 20 rats/group). **A** Body weight during rat modeling. **B** Body weight of rats after modeling (on the 21st day). **C** Body weight of rats during treatment. **D** Body weight of rats 14 days after treatment. Error bars represent standard deviation. ## *P* < 0.01 vs. CON group; ** *P* < 0.01 vs. LE group

There was no significant difference in the initial body weight of rats among the three groups (*P* > 0.05). During the modeling period, the body weight of rats in the letrozole-treated groups increased significantly faster than that of rats in the CON group (Fig. 3A). After modeling, the rats in the modeling groups weighed significantly more than the CON rats (*P* < 0.01; Fig. 3B). However, EA significantly slowed the increase in the body weight during the 2 weeks of treatment (Fig. 3C). The final body weight of rats in the EA group was significantly lower than that of rats in the LE group (*P* < 0.01; Fig. 3D).

### Ovary weight and ovarian morphological changes

**Fig. 4:**
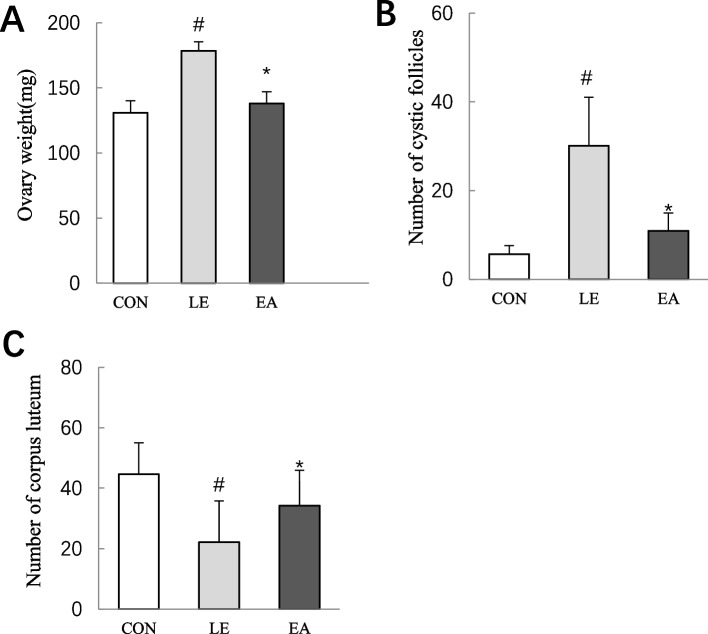
Rat ovary weight and evaluation of follicles in ovaries of rats (*n* = 10 rats/group). **A** Ovary weight of rats after treatment. **B** Number of cystic follicles in ovaries of rats; **C** Number of luteal corpus in ovaries of rats; Error bars represent SD. # *P* < 0.05 vs CON group. * *P* < 0.05 vs LE group

Compared with the CON group, the ovary weight of rats in the LE group increased significantly (*P* < 0.05; Fig. 4A), which is in line with the characteristics of PCOS. However, the ovary weight of rats in the EA group was significantly lower than that of rats in the LE group (*P* < 0.05; Fig. 4A).

**Fig. 5:**
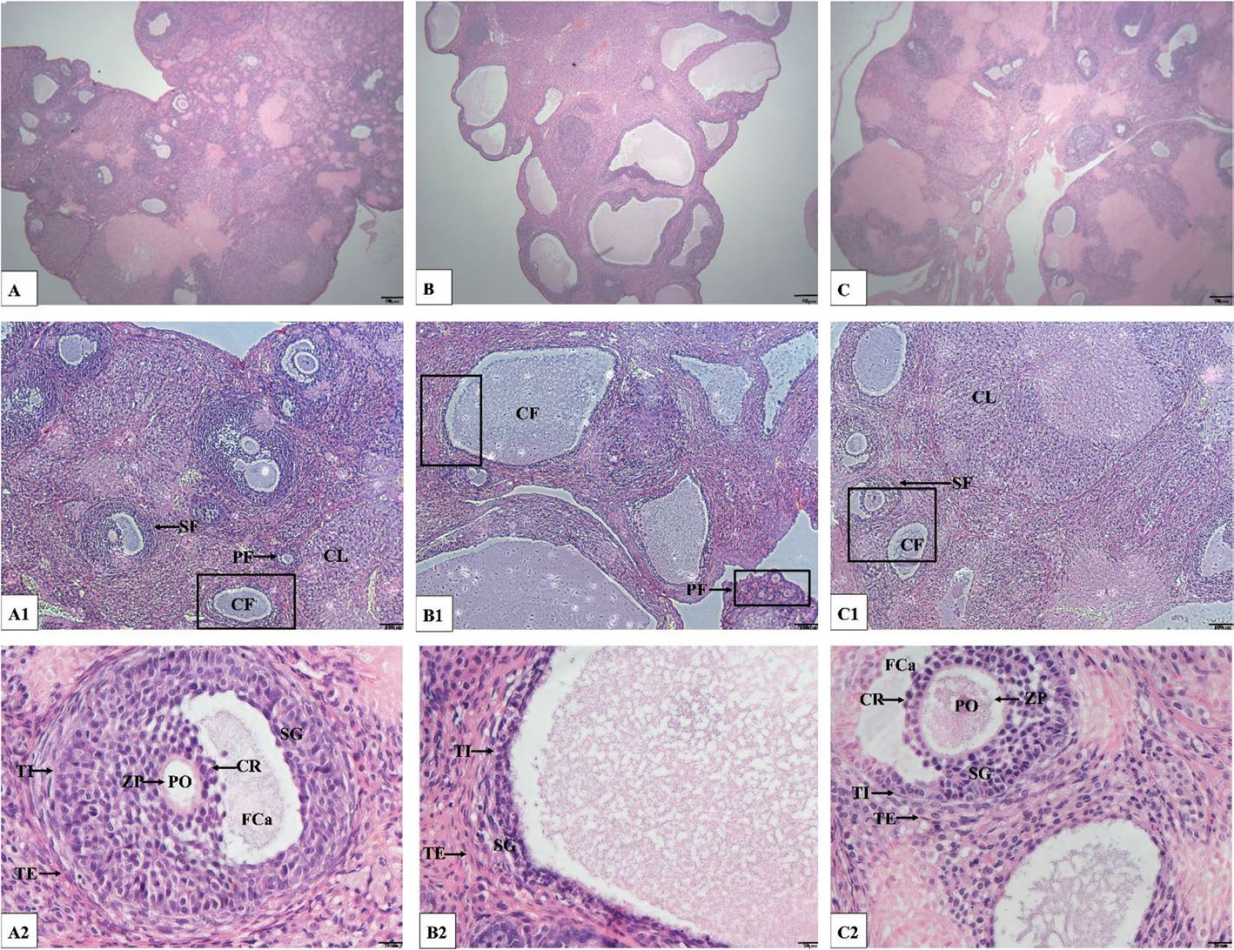
Morphological appearance in ovaries of rats from three groups (n = 10 rats/group). A-C) Survey view showing ovaries, A-C represents the CON group, LE group and EA group respectively (magnification40×; distance bars, 50 μm); A1-C1) Higher magnification image of A-C. (magnification 100×; distance bars, 100 μm); A2-C2), Magnification 400×; distance bars, 20 μm. A2) Higher-power view of rectangular area in A1 shows a secondary follicle with corona radiata and zona pellucida, the granulosa cell layer(GL) and theca cell layer (TL) appear normal; B2) Higher-power view of rectangular area in B1 shows a cystic dilated follicle. The cyst wall has a thick, vascularized layer of luteinized cells and thin granulosa cell layer; C2) Higher-power view of rectangular area in C1 shows a cystic follicle and a secondary follicle. PF, primary follicles. SF, secondary follicle. CF, cystic follicle. CL, corpus luteum. GL, granulosa cell layer. TL, theca cell layer. ZP, zona pellucida. CR, corona radiata. PO, primary oocyte

The ovaries of rats in the CON group were ruddy in appearance, and follicles at different stages were observed under the microscope. The granulosa cells were densely arranged, mostly in 7–8 layers, showing structures such as cumulus and corona radiata (Fig. 5A-A2). Compared with the CON group, Granulosa cells were loosely arranged in LE group rats, the follicular oocytes and radial crowns of LE group rats disappeared, number of cystic follicles increased (*P* < 0.05; Figs. 4B and 5B-B2), and number of corpora lutea decreased (*P* < 0.05; Figs. 4C and 5B-B1). Compared with the LE group, the number of ovarian cystic follicles decreased (*P* < 0.05; Figs. 4B and 5C-C1), number of corpora lutea increased (*P* < 0.05; Figs. 4C and 5C-C1), and a small number of normally developed follicles appeared (Fig. 5C-C1) in the EA group rats.

### Circulating hormone levels

**Fig. 6:**
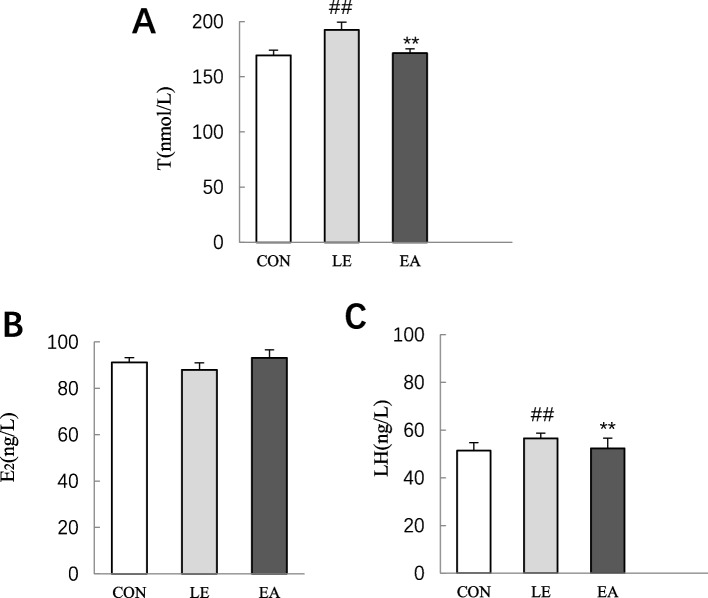
Comparison of testosterone, estradiol, and luteinizing hormone levels in the serum of rats from the CON, LE, and EA group (*n* = 10 rats/group). **A** Serum testosterone concentration. **B** Serum estradiol concentration. **C** Serum luteinizing hormone concentration. Error bars repressent standard deviation. ## *P* < 0.01 vs. CON group; ** *P* < 0.01 vs. LE group

Compared with the CON group, the levels of serum T and LH in the LE group were significantly increased (*P* < 0.01; Fig. 6A and C). After EA treatment, the levels of T and LH in the EA group significantly decreased than those in the LE group (*P* < 0.01; Fig. 6A and C). The level of E_2_ was not significantly different among the groups (*P* > 0.05; Fig. 6B).

### Kisspeptin, GnRH, Dyn, and NKB levels in the hypothalamic ARC of rats

**Fig. 7:**
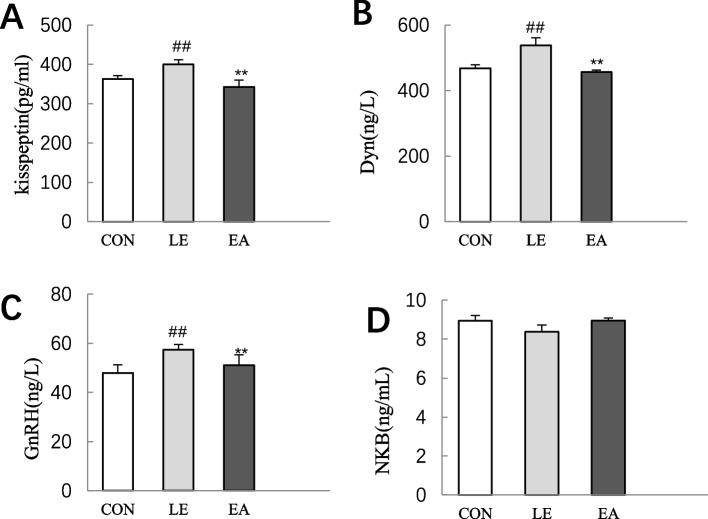
Comparison of the concentration levels of different neuropeptides (kisspeptin, GnRH, Dyn, and NKB) in the hypothalamic arcuate nucleus of rats from the CON, LE, and EA groups (*n* = 10 rats/group). Error bars represent standard deviation. ## *P* < 0.01 vs. CON group; ** *P* < 0.01 vs. LE group

KNDy neurons dominate the secretion of GnRH via the binding of kisspeptin to Kiss1r on GnRH neurons. They also secrete NKB and Dyn neuropeptides to jointly regulate GnRH secretion. Unlike kisspeptin, which promotes the release of GnRH, Dyn inhibits GnRH release via the feedback regulation of KNDy neurons in an autocrine or paracrine manner, whereas NKB regulates the pulsatile release of GnRH by affecting the secretion of kisspeptin [41] and Dyn [42]. In the present study, the levels of the three neuropeptides secreted by KNDy neurons were assessed. Compared with the CON group, the level of kisspeptin, GnRH, and Dyn in the hypothalamic ARC of rats in the LE group was significantly increased (*P* < 0.01; Fig. 7A–C), and compared with the LE group, the level of kisspeptin, GnRH, and Dyn in the EA group was significantly decreased (*P* < 0.01; Fig. 7A–C). There was no significant difference in the level of NKB among the three groups (*P* > 0.05; Fig. 7D).

### Coexpression of AR and ERα in ARC *Kiss1* cells

KNDy neurons receive sex hormone feedback signals via the respective sex hormone receptors expressed on themselves [43–45]. Thus, the triple-label immunofluorescence assay was performed to detect the coexpression of AR and ERα in the KNDy neurons of rats in the three groups. *Kiss1* encodes kisspeptin, which was used to label KNDy neurons.

**Fig. 8:**
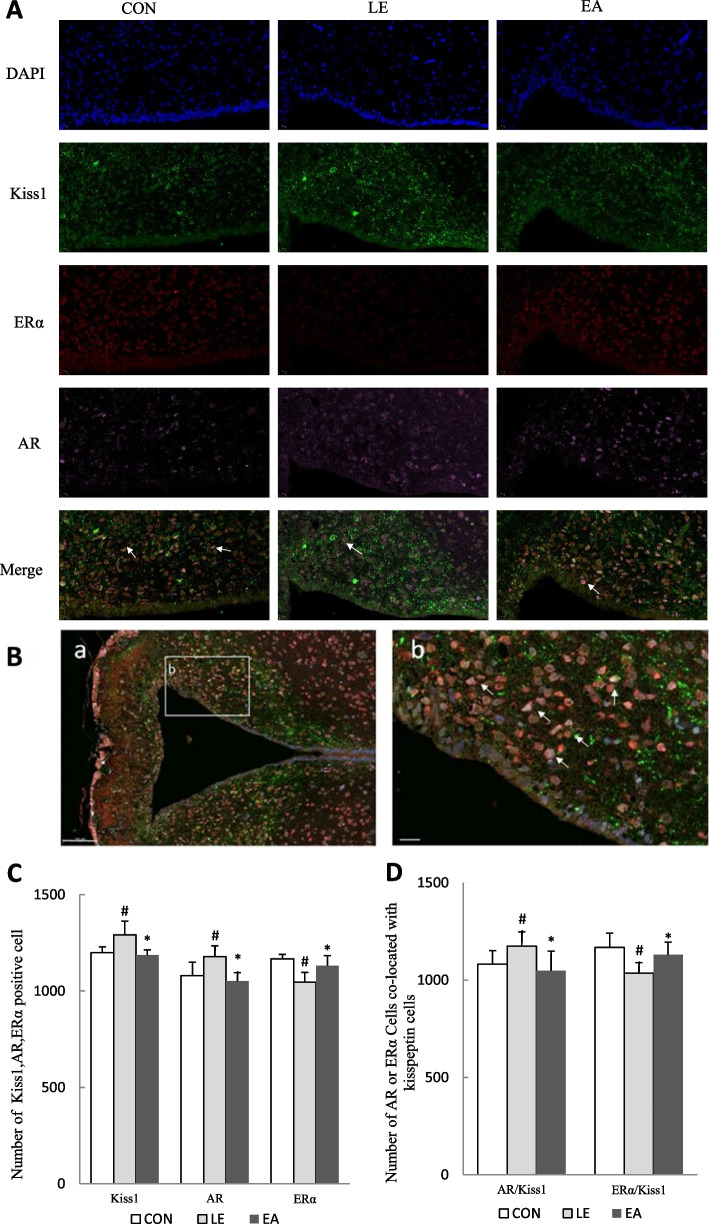
Coexpression of AR, ERα, and Kiss1 in the hypothalamus arcuate nucleus (ARC) of rats from the CON, LE, and EA groups (*n* = 5 rats/group). **A** The coexpression of AR, ERα, and Kiss1 in the hypothalamic ARC of rats was assessed via triple-label immunofluorescence (400×). Scale bars: 50 μm. **B** a: A lower magnification image showing the entire ARC and median eminence (150×). Rectangular frame represents ARC analyzed in **A**.3v:Scale bar: 100 μm. b: A higher magnification image showing the rectangle area (400×), Scale bars: 20 μm. **C** The number of AR-, ERα-, and Kiss1-positive cells in the hypothalamic ARC of rats in each group. **D** The number of AR- and ERα-positive cells coexpressed with Kiss1 cells in the hypothalamic ARC of rats in each group. Values shown are mean ± standard deviation. Error bars represent standard deviation. # *P* < 0.05 vs. CON group; * *P* < 0.05 vs. LE group

After fluorescent staining, Kiss1-positive cells were marked in green, AR-positive cells were marked in pink, and ERα-positive cells were marked in red (Fig. 8A and B). The triple-label fluorescence images showed that AR and ERα cells colocalized on the KNDy neurons of ARC (Fig. 8A and B). Compared with the CON group, the number of Kiss1- and AR-positive cells as well as AR/Kiss1-positive cells in the LE group increased (*P* < 0.05) and the number of ERα- and ERα/Kiss1-positive cells decreased (*P* < 0.05; Fig. 8C and D). Compared with the LE group, EA treatment reversed the increase in Kiss1-, AR-, and AR/Kiss1-positive cells and increased the number of Erα and ERα/Kiss1-positive cells (*P* < 0.05; Fig. 8C and D).

### Expression of Kiss1, Kiss1r, AR, and ERα proteins in the hypothalamic ARC of rats

**Fig. 9:**
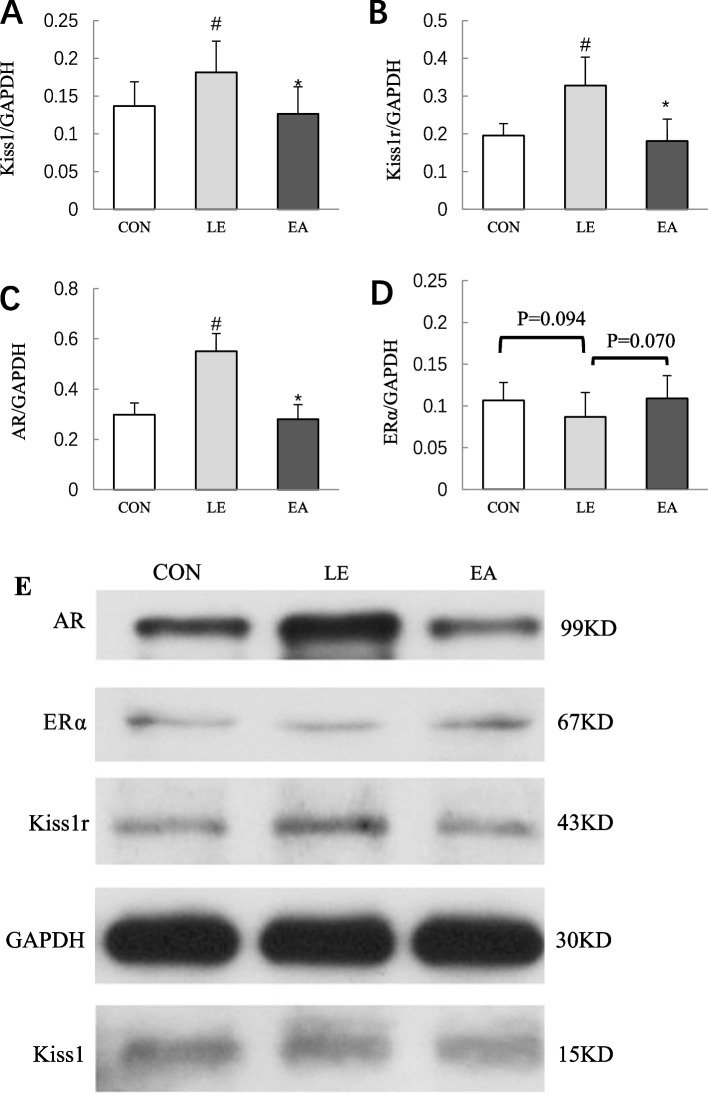
Protein expression levels of Kiss1, Kiss1r, AR, and ERα in the hypothalamic arcuate nucleus (ARC) of rats from the CON, LE, and EA groups (*n* = 5 rats/group). **A**–**D** The bar graph showing the ratio of Kiss1, Kiss1r, AR, and ERα band intensity to GAPDH band intensity in the ARC of rats from three groups. **E** Representative western blot of Kiss1, Kiss1r, AR, ERα, and GAPDH; Error bars represent standard deviation. # *P* < 0.05 vs. CON group; * *P* < 0.05 vs. LE group

Compared with the CON group, the expression of Kiss1, AR, Kiss1r in the hypothalamic ARC nucleus of rats in the LE group significantly increased (*P* < 0.05), and EA treatment reversed the increase in Kiss1, Kiss1r, and AR expression (*P* < 0.05; Fig. 9A–C). Compared with the CON group, the expression of ERα in the LE group exhibited a downward trend and the EA group exhibited an upward trend; however, the difference among the groups was not statistically significant (Fig. 9D). This might be attributed to the small sample size. The bar graph in Fig. 9 shows the levels of expression relative to that of actin bands in rat ovarian tissue from the three groups.

### mRNA expression of *Kiss1*, *Kiss1r*, *Ar*, and *Esr1* in the hypothalamic ARC of rats

**Fig. 10:**
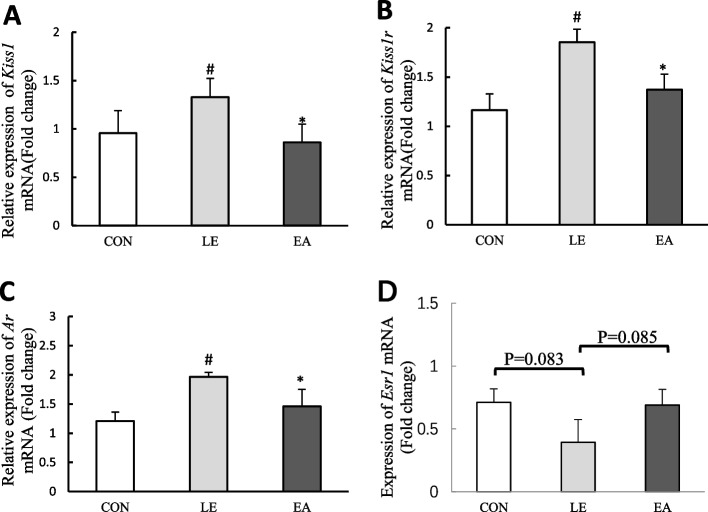
mRNA expression levels of *Kiss1*, *Kiss1r*, *Ar* and *Esr1* in the hypothalamic arcuate nucleus of rats from the CON, LE, and EA groups (*n* = 5 rats/group). Error bars represent standard deviation. #*P* < 0.05 vs. CON group; *P < 0.05 vs. LE group

Compared with the CON group, the mRNA expression of *Kiss1*, *Kiss1r*, and *Ar* in the hypothalamic ARC of rats in the LE group significantly increased (*P* < 0.05), and EA treatment reversed the increase in *Kiss1*, *Kiss1r*, and *Ar* expression (*P* < 0.05; Fig. 10A–C). Compared with the CON group, the mRNA expression of *Esr1* in the LE group exhibited a downward trend and the EA group exhibited an upward trend; however, the difference among the groups was not statistically significant (Fig. 10D).

## Discussion

Acupuncture, as a characteristic TCM therapy, has been shown to be effective in PCOS treatment [5–9]. However, the exact mechanism of acupuncture treatment in PCOS remains unclear. Most studies explored the mechanism of acupuncture from the perspective of peripheral organs or tissues (such as ovary and skeletal muscle) [46, 47]. For example, EA improved PCOS-like traits by enhancing autophagy in rats and regulating the lipid metabolism regulator SREBP1 to improve insulin resistance, mitochondrial dysfunction, and endoplasmic reticulum stress [46, 47]. Moreover, low-frequency EA reduced ovarian androgen synthase expression, increased aromatase expression [48], regulated local ovarian factor and gonadotropin receptor expression [38, 49], and enhanced insulin sensitivity in PCOS rats [50]. A study on the central mechanisms of the effect of EA showed that EA could regulate the gonadal axis by adjusting the secretion of β-EP [51] and expression of *μ* and *κ receptor* mRNA in the hypothalamus of rats [52].

Although defined by ovarian dysfunction, long-standing and more recent pieces of evidence suggest that androgen activity in the brain plays a key role in the development and pathophysiology of PCOS [34, 35]. GnRH is regulated by many signals, among which kisspeptin is the most effective stimulator, and KNDy neurons located in ARC are mainly involved in regulating the basal pulsatile release of GnRH and are the target cells of steroid hormone negative feedback [30].

Compared with normal rats, the letrozole-induced PCOS model rats exhibit acyclicity, elevated serum LH and T levels. Ovaries from LE groups showed high incidence of subcapsular ovarian cyst and capsular thickening together with incomplete luteinization and decreased number of corpora lutea. These traits correspond to human PCOS. Some studies showed that ARC kisspeptin positive cells and *Kiss-1* gene expression increased in LE rats [33, 53], which indicates enhanced KNDy neuron activity. Compared with the letrozole model, in a neonatally androgenized female rat model, because T was administered prior to the differentiation of hypothalamus and pituitary cells, these organs were rendered nonresponsive to steroids, GnRH, and follicle-stimulating hormone [54]. Therefore, in the present study, the letrozole model was used to explore the effect of EA on the kisspeptin-GnRH/LH neural circuit. In a previous study, the efficacy of different acupoints on hyperandrogenism among four groups (ST36, SP6, CV4, and comprehensive groups), and the results showed that the CV4 group had the best therapeutic effect [55]. In the present study, therefore, acupuncture at the CV4 point was chosen as the treatment site.

The results of this study revealed that EA improved the estrous cycle and ovarian polycystic morphology of letrozole-treated rats and reduced their elevated serum LH and T levels, which is consistent with a previous study result [38]. The results also showed that the level of GnRH and expression of kisspeptin and Kiss1r were increased in the hypothalamic ARC of PCOS rats. EA reversed the elevated GnRH level and the protein and mRNA expressions of *Kiss1* and *Kiss1r*. This indicated that the change of kisspeptin-GnRH/LH neural circuit is similar in different PCOS models, and EA could ameliorate the hyperactive GnRH/LH pulse, possibly by inhibiting the kisspeptin/ Kiss1r system.

Increase in the levels of kisspeptin and Dyn in the hypothalamic ARC of the rats in the LE group indicated that the balance of the three neuropeptides secreted by KNDy neurons in PCOS rats was disrupted; however, EA improved this imbalance. Preclinical PCOS-like animal models demonstrated considerable heterogeneity regarding alterations in kisspeptin neuron anatomy and function owing to the method through which the model was established [56]. The results of different studies on changes in NKB expression in PCOS models are inconsistent. Most studies revealed increased NKB protein or gene expression [30, 33, 57], but other studies showed no significant changes in NKB [39], in line with the results of the current study. These differences may be attributed to the different detection and modeling methods. The KNDy model proposes that the stimulatory role of NKB and inhibitory role of Dyn are crucial for the coordination of kisspeptin secretion in regulating the pulsatile release of GnRH and the subsequent downstream gonadotropin release. Therefore, further studies are needed to uncover the interaction of these three peptides.

In the current study, the benign regulatory effect of EA on the function of KNDy neurons was validated in LE rats. KNDy neurons act as GnRH pulse generator and are regulated by sex hormones [24, 58]; both AR and ER participate in the regulation of *Kiss-1* mRNA through receptors expressed in KNDy neurons [43]. Therefore, the effect of EA on the expression of AR and ER on KNDy neurons was next evaluated. Triple-label fluorescence and western blotting detection results demonstrated that the expression of sex hormone receptors on KNDy neurons was abnormal in PCOS rats. Excessive AR expression suggested an enhanced AR-mediated effect and deficient ERα expression suggested a decreased sensitivity of KNDy neurons to E_2_ negative feedback, which are likely the factors that cause KNDy neuron dysfunction in PCOS rats. EA can reduce elevated AR expression in LE rats, suggesting that EA not only decreases the synthesis and weakens its effect in the periphery but also contributes to the attenuation of androgen activity in the brain. The upregulation effect of EA on the expression of ERα detected in the triple-labeled fluorescence experiment was not significant in the western blot results, which may be attributed to the small sample size, but the results still revealed a trend of upregulation. These results suggested that EA is helpful in restoring the requirement of normal E_2_ feedback. This finding will be validated in a future study.

Finally, the letrozole-induced dysfunction of the kisspeptin-GnRH/LH neural circuit in PCOS rats was identified. EA improved the kisspeptin-GnRH/LH neural circuit abnormalities in PCOS rats, possibly via the benign regulation of AR expression in KNDy neurons. There are some limitations in this study, including the lack of evaluation of the androgen signaling pathway and the interaction of other neurons (e.g., interaction of GABAergic and agouti-related protein/neuropeptide Y neuron with KNDy neurons) that regulate GnRH neuron functions. Thus, the effect of EA on these factors should be studied in the future.

## Conclusion

Taken together, improvement in the reproductive hallmarks of PCOS rats via EA may be achieved through the regulation of the brain kisspeptin-GnRH/LH neural circuit by attenuating androgen activity. Thus, the results provide an experimental basis for acupuncture as an adjuvant medical therapy on PCOS.

## Data Availability

The datasets used and/or analyzed in the current study are available from the corresponding author upon reasonable request.

## Abbreviations

PCOS: Polycystic ovary syndrome
AR: Androgen receptor
ER: Estrogen receptor
ARC: Arcuate nucleus
T: Testosterone
E2: Estradiol
LH: Luteinizing hormone
FSH: Follicle-stimulating hormone
GnRH: Gonadotropin-releases hormone
Dyn: Dynorphin
NKB: Neurokinin B
KNDy: Kisspeptin/Neurokinin B/Dynorphi
Kiss1r: Kisspeptin receptor
CMC: Sodium carboxymethyl
LE: Letrozole
EA: Electroacupuncture
CV4: Guan Yuan acupoints
TCM: Traditional Chinese Medicine
Elisa: Enzyme-linked immunosorbent assay
RT-PCR: Reverse Transcription-Polymerase Chain Reaction
